# The relationship between anti-HPV-16 IgG seropositivity and cancer of the cervix, anogenital organs, oral cavity and pharynx, oesophagus and prostate in a black South African population

**DOI:** 10.1186/1750-9378-2-6

**Published:** 2007-03-02

**Authors:** Freddy Sitas, Margaret Urban, Lara Stein, Valerie Beral, Paul Ruff, Martin Hale, Moosa Patel, Dianne O'Connell, Xue Qin Yu, Anke Verzijden, Dianne Marais, Anna-Lise Williamson

**Affiliations:** 1 Cancer Epidemiology Research Group, National Health Laboratory Service, P.O.Box 1038, Johannesburg, 2000, South Africa; 2 Research Division, The Cancer Council NSW, P.O. Box 572, Kings Cross, 1340, NSW, Australia; 3 Schools of Public Health, Universities of Sydney and NSW, Sydney, Australia; 4 Epidemiology Unit, Cancer Research UK, Oxford, UK; 5 Faculty of Health Sciences, University of the Witwatersrand, Johannesburg, South Africa; 6 Anatomical Pathology Department, National Health Laboratory Service, Johannesburg, South Africa; 7 Dept. of Epidemiology & Biostatistics, Radboud University Nijmegen Medical Centre, Nijmegen, The Netherlands; 8 Division of Medical Virology, Institute of Infectious Disease and Molecular Medicine, University of Cape Town Medical School, Observatory, Cape Town, South Africa

## Abstract

**Background:**

Human papillomavirus type 16 (HPV-16) infection is an important cause of cervical cancer, other anogenital cancers and, possibly, some oral and pharyngeal cancers. The association of HPV-16 with oesophageal and with prostate cancers has not been firmly established.

**Methods:**

We analysed sera from 3,757 HIV seronegative black South Africans using an anti-HPV IgG enzyme-linked immunosorbent assay (ELISA). The subjects were recruited from 1995 to 2000 as part of an ongoing cancer case control study. Cases were patients with newly diagnosed cancers of the cervix (n = 946), other anogenital organs (n = 80), the oral cavity and pharynx (n = 102), the oesophagus (n = 369) or the prostate (n = 205). The comparison group consisted of 2,055 age and sex-matched patients randomly selected from the same data base, diagnosed at the same hospitals, but with a vascular disease or with a cancer unrelated to HPV infection. Subjects' sera were randomly and blindly allocated onto ELISA plates. Optical density (OD) levels of anti-HPV-16 IgG of > 0.45 and ≥ 0.767 were taken to be cut-offs for negative, medium and high antibody levels.

**Results:**

After adjustment for potential confounders, cancer types that showed a statistically significant association with increased anti-HPV-16 IgG antibody (Ab) levels were cancer of the cervix (OR for medium Ab levels = 1.6, and for high = 2.4, p < 0.0001), cancers of other anogenital organs (OR for medium or high Ab levels = 2.5, p = 0.002), and cancer of the oesophagus (OR for medium Ab = 1.3, and high Ab levels = 1.6 p = 0.002). Cancers of the oral cavity and pharynx showed a borderline significant association in the unadjusted model (p = 0.05) but after adjustment for confounding the trend in relation to Ab levels was positive but not statistically significant (OR for medium Ab = 1.1, and high Ab = 1.5 p = 0.13). Prostate cancer was not associated with HPV-16 seropositivity (OR for medium Ab level = 1.4, and for high Ab level = 1.3, p = 0.3).

**Conclusion:**

If there is indeed an association between HPV-16 and oesophageal and possibly also some oral cavity and pharyngeal cancers, then emerging HPV vaccines may also reduce, at least in part, the incidence of these leading cancer types.

## Background

Cancers of the cervix, oesophagus, oral cavity and prostate gland are among the leading cancer types in South Africa[1]. Infection with specific oncogenic human papillomaviruses (HPV) such as HPV-16 is regarded as a necessary cause of cervical and other anogenital cancers [2] and has also been associated with cancers of the oral cavity and especially the pharynx [3]. HPV-16 infection has been linked to squamous cell carcinoma of the oesophagus in several epidemiological studies and clinical series [4-6], but not in others [7]. Likewise, prostate cancer has been associated with certain HPV genotypes in some studies [8-10], but not in others [11,12].

The aim of this study was to assess, in a standardised manner, the known association between anti-HPV-16 IgG antibodies and cancers of the cervix and anogenital region and to explore the possible associations between anti-HPV-16 IgG serological responses and cancers of the oral cavity and pharynx, oesophagus and prostate. Measuring the presence and viral loads of various HPV DNA genotypes in the target organs would have been the method of choice, but collecting tissue from cases and controls is not practical in large-scale epidemiological studies. Thus serological markers of HPV infection were used.

## Materials and methods

### Recruitment of cases and controls

An ongoing case-control study on lifestyle factors, infection and cancer began in 1995 in the main public referral hospitals for cancer treatment in greater Johannesburg, South Africa. Infectious agents already tested include HIV [13], human herpes virus 8[14], and human herpesviruses 1 to 6 [15]. All newly diagnosed black African adults with cancer are eligible for inclusion. Patients are asked whether they have previously had a cancer and, where possible, this is verified by checking records. In about 80% of cases the diagnosis and primary site were confirmed by histology, haematology, or cytology [13]. During the time period for this study (1995 – 2000) selected patients from the same hospitals with a new diagnosis of cardiovascular disease were also included. Following written or witnessed oral informed consent, patients were interviewed by trained nurses using a two-page demographic and lifestyle questionnaire[16]. Interviews were conducted in the main vernacular languages. Blood specimens were collected prior to initiation of treatment. Sera were separated, aliquotted and stored at minus 30°C.

Sera from subjects were batch tested for HIV-1 by the South African National Health Laboratory Service Serology Laboratory using standard serological methods described previously [13]. The current analysis used questionnaire data and serum samples from 3757 HIV seronegative subjects in order to determine the relative importance of HPV-16 in relation to risk of a number of cancer types. Cases were subjects with cancer of the cervix (n = 946), other anogenital organs (61 females, 19 males, comprising 10 anal, 51 vulval, 6 vaginal and 13 penile cancers), cancers of the oral cavity and pharynx (21 females, 81 males, comprising 20 cancers of the tongue, 25 of the floor of mouth, 8 of the gum, 10 of the parotid/salivary glands, 6 of the tonsil, and 11 of the oro-naso pharynx, and 22 cancers in other regions of the oral cavity), cancer of the oesophagus (126 female, 243 males) and cancer of the prostate (205 males). The comparison group consisted of 2,055 age and sex-matched patients randomly selected from the same data base, diagnosed at the same hospitals, but with a vascular disease or with a cancer type thought to be unrelated to HPV infection. These comprised 1382 female and 673 male subjects with breast cancer (506 females), colorectal cancer (60 females, 51 males), endocrine cancer (19 females, 9 males), Hodgkin lymphoma (37 females, 37 males), leukaemia (104 females, 103 males), liver cancer (9 females, 13 males), lung cancer (34 females, 99 males), myeloma (53 females, 65 males), non-Hodgkin lymphoma (27 females, 40 males), stomach cancer (27 females, 36 males), other minor cancer types (132 females, 127 males) and with cardiovascular disease (374 females, 93 males).

### HPV-16 serology

Serum aliquots from all participants were air freighted in dry ice to the University of Cape Town, Division of Medical Virology for anti-HPV-16 IgG testing by ELISA. The ELISA was a modification of that described by Studentsov and colleagues [17,18], using polymer solutions (Sigma) for blocking (polyvinyl alcohol, PVA) and secondary antibody (polyvinylpyrrolidone, PVP) enhancement. This enhanced ELISA uses serum at a 1:100 dilution instead of a previous 1:20 dilution [19]. Plates were coated with 100 μl of 0.05 μg virus-like particles (VLP) to HPV-16, (MedImmune Inc) in phosphate buffer solution (PBS) overnight at 4°C. After washing twice in PBS, plates were blocked with 200 μl 0.5% PVA in PBS (0.5% PVA) at room temperature. After washing six times in PBS, serum diluted 1:100 in 0.5% PVA was added and allowed to incubate at 37°C for one hour. The subsequent procedure used 0.8% PVP and 0.5% PVA in PBS for the dilution of the horse radish peroxidase (HRP) conjugated anti-human IgG with six PBS washes between this and other procedures [19].

### Minimising laboratory variation

To minimise laboratory variation and case-control bias, serum aliquots from the cases and the comparison group were randomly allocated on each ELISA plate by five age groups (25–34, 35–44, 45–54, 55–64, and 65–74 years) and by case-comparison status. Specimens from males and females were run on separate plates. The laboratory staff were 'blinded' with regard to the distribution of the aliquots on the plates. In addition, for quality control purposes, ten adult consenting volunteer donors provided anonymous blood samples to use as plate controls. These donors were tested for anti-HPV-16 IgG antibody responses prior to the commencement of this study, and the sera of three donors who were found to have low, medium and high anti- HPV-16 IgG optical density values were added on wells on each of the 111 ELISA plates. As a secondary precaution, pooled sera of children collected for other purposes were added on wells of each ELISA plate to represent an additional reference group. Children's anti-HPV IgG titres are usually low, and therefore could represent a true negative (uninfected) group [2]. Intra-class correlation coefficients were estimated in the volunteer and children's sera to measure the plate-to-plate assay variability.

### Determination of cut-off values for negative, medium and high antibody levels

The ELISA optical density (OD) cut-off for positivity was 0.45, determined by calculating the mean plus 4 standard deviations (SD) of the absorbance values obtained from children's serum samples tested by VLP-16 IgG ELISA [20]. OD values between 0.45 and 0.767 were considered medium antibody levels and OD values greater than 0.767 were considered as high. The value of 0.767 was the median OD value in the seropositive comparison group. Due to small numbers, cancers of the male and female anogenital organs (aside from cervix) were only divided into HPV-16 IgG seropositives (OD ≥ 0.45) and seronegatives (OD <0.45).

### Statistical methods

In the comparison group, bivariate, chi-squared and t-statistics were calculated to measure the association between anti-HPV-16 IgG optical density and a number of demographic and lifestyle factors, in males and females, such as age (18–34, 35–54 and 55–74 years), years of education (less than 4, 4 to 6, 7 to 9 and 10 or more years), lifetime number of sexual partners (0 to 1, 2 to 4, 5 or more), residence (urban/rural), birthplace (urban/rural), alcohol consumption (ever/never), tobacco smoking (never, ex-, current), and parity (0 to 2, 3 to 5 and 6 or more children), all of which were found in some studies to be associated with HPV acquisition and with the disease conditions of interest [2]. In the analysis of cancers of male and female anogenital organs a variable containing sex and parity was included in the model (males, females with 0–2, with 3–5 and with 6 or more children). To estimate the prevalence of the HPV-16 IgG seropositivity in the cases and the comparison group (subjects with other cancer types or with vascular disease), logistic regression was used, adjusted for age, sex and number of sexual partners, using females with 0–1 sexual partners, aged 18–34 years as the reference. The odds and 95% confidence intervals, were transformed into proportions (seropositivity) by the formula odds/1+odds. The distribution of HIV in relation to a number of socio-demographic variables in the comparison group is shown in table 1.

**Table 1:** Distribution of anti-HPV-16-IgG seropositivity in the comparison group

	**Females**				**Males**			
	**N**	**% HPV +ve (>0.45)**	p *,#	**Mean HPV-16-OD**	**N**	**% HPV +ve (>0.45)**	P *, #	**Mean HPV-16 OD**

**Age (years)**								
18–34	113	67.3	0.004*	0.73	76	42.1	0.003*	0.53
35–54	679	66.0		0.76	315	57.8		0.63
55–74	590	58.6		0.65	282	61.3		0.63

**All ages**	**1382**	**63.0**			**673**	**57.8 **		

**Years of Education**								
<4	264	64.8	0.8*	0.71	140	64.3	0.1*	0.70
4–6	224	58.0		0.69	135	56.3		0.62
7–9	529	63.1		0.70	220	60.9		0.63
10 and +	359	64.1		0.73	158	48.7		0.53
Missing	6	-		-	20	-		-
**Number of sexual partners**								
0–1	261	57.9	0.01*	0.66	57	50.9	0.1*	0.56
2–4	834	63.1		0.69	286	55.6		0.64
5+	260	68.1		0.82	299	61.2		0.61
Missing	27	-		-	31	-		-
**Current residence**								
Urban	1154	62.4	0.2#	0.71	557	56.6	0.05#	0.61
Rural	207	67.1		0.72	94	67.0		0.70
Missing	21	-		-	22	-		-
**Place of birth**								
Urban	735	64.6	0.2#	0.74	318	56.0	0.2#	0.58
Rural	646	61.0		0.68	338	60.4		0.66
Missing	1	-		-	17			-
**Alcohol consumption**								
Ever	726	64.7	0.3#	0.73	516	57.6	0.5#	0.61
Never	651	61.1		0.69	139	59.7		0.66
Missing	5	--		-	18	-		-
**Smoking**								
Current	92	69.6	0.5*	0.79	218	58.7	0.8*	0.60
Ex-	184	59.8		0.70	260	58.5		0.56
Never	1094	63.0		0.71	179	55.9		0.64
Missing	12	-		-	16	-		-
**Parity**								
0–2	510	66.9	0.1*	0.77	-	-	-	-
3–5	607	60.3		0.68	-	-	-	-
6+	254	61.0		0.67	-	-	-	-
Missing	11	-	-	-	-	-	-	-

P-values are either tests for trend* or homogeneity#. P-values for variables other than age are age-adjusted.

Odds ratios to compare anti-HPV-16 IgG levels in patients with each cancer type of interest (cervical, other anogenital, oral and pharyngeal, oesophageal, and prostate) and the comparison group were calculated using unconditional logistic regression and shown in table 2. For each cancer type of interest, odds ratios were first adjusted for age and sex and then further adjusted for age group, sex (only for cancers of the oral cavity and pharynx, oesophagus and other anogenital organs) and also for a number of additional confounders which were thought to be important in the aetiology of cervical (number of sexual partners, smoking, parity and hormonal contraception [21,22], other anogenital (smoking, parity, number of sexual partners-in keeping with cervical cancer) oral cavity and pharynx (smoking, alcohol and number of sexual partners [3]), oesophageal (smoking, alcohol [13]), and prostate cancer (number of sexual partners [8-10]). We adjusted for education (as a proxy of socio-economic condition) and for birthplace and current residence (to take account for possible geographical differences in cancer incidence and referral) in all the models. A third, parsimonious model, was obtained by removing variables from the full model and monitoring the effect on the odds ratio for HPV infection. Results are summarised in Table 2. Missing values were included wherever possible as an additional category for each variable in the models. The number of cases of prostate cancer aged 18–34 was too small for proper adjustment using logistic regression so these were excluded. All p values are 2-tailed.

**Table 2:** Risk of developing selected cancers in relation to anti-HPV-16 IgG OD levels

**Cancer site or type**	**HPV-16 OD status**	**Comparison group (N)**	**Cases (N)**	**OR**_**1 **_**(95% CI)**	**Adj. OR**_**2 **_**(95% CI)**	**Adj. OR**_**3 **_**(95% CI)**
**Cervix (women only)**						
	Low (Negative)<0.45	512	201	1	1	1
	Medium 0.45–0.767	411	273	1.71	1.50(1.18–1.90)	1.64(1.30–2.08)
	High >0.767	459	472	2.63	2.38 (1.91–2.97)	2.50(2.01–3.11)
	*P-trend*			*<0.0001*	<0.0001*	*<0.0001*^†^
**Anogenital organs (men and women)**						
	Low <0.45	798	15	1	1	1
	Medium or high > = 0.45	1257	65	2.69	2.30(1.28–4.11)	2.49(1.40–4.43)
	P=			0.0007	0.005**	*0.002*^††^
**Oral cavity and pharynx (men and women)**						
	Low (Negative)<0.45	798	33	1	1	1
	Medium 0.45–0.767	625	32	1.18	1.13(0.66–1.91)	1.09(0.64–1.83)
	High >0.767	632	37	1.65	1.49(0.89–2.51)	1.47(0.88–2.47)
	P-trend			0.05	0.13***	*0.13*^†††^
**Oesophagus (men and women)**						
	Low (Negative)<0.45	798	112	1	1	1
	Medium 0.45–0.767	625	123	1.33	1.25(0.92–1.69)	1.26(0.94–1.70)
	High >0.767	632	134	1.65	1.53(1.14–2.07)	1.59(1.19–2.13)
	*P-trend*			*0.0006*	0.0005***	*0.002*^††††^
**Prostate (men only)**						
	Low (Negative)<0.45	286	65	1	1	1
	Medium 0.45–0.767	214	81	1.39	1.33(0.87–2.04)	1.39(0.93–2.09)
	High >0.767	173	59	1.33	1.22(0.77–1.93)	1.33(0.86–2.07)
	*P-trend*				0.35****	*0.18*^†††††^

Notes: Adjustment factors

**OR**_1_: Age and sex (where appropriate)

**OR**_2_:

* Age group, education, parity, number of sexual partners, birthplace, current residence and smoking

** Age group, education, parity and sex (see methods), number of sexual partners, birthplace, current residence and smoking.

*** Age group, sex, education, birthplace, current residence, alcohol usage and smoking.

**** Age group, education, birthplace, current residence and number of sexual partners.

**OR**_3_:

^† ^Education, parity, number of sexual partners and smoking

^†† ^Sex, smoking, parity

^††† ^Age group, sex, smoking, current residence, education

^†††† ^Age group, sex, smoking, education

^††††† ^Age group, sex, smoking, education

## Results

The anti-HPV-16 IgG levels of pooled children's sera were indeed low (mean OD = 0.015, SD = 0.012) compared to the mean antibody level of the (adult) study subjects (mean OD = 0.825, SD = 0.635). To measure the reproducibility of the ELISA assay between plates, the intra-class correlation coefficient was calculated for the high, medium and low anti-HPV IgG levels of the volunteer sera and was found to be excellent (ρ = 0.9724).

Using an optical density cut-off of 0.45, the HPV-16 IgG seropositivity in the comparison group was 63% in females and 58% in males (age adjusted χ^2 ^= 5.34, p = 0.02). In females, there was a significant inverse trend in the HPV-16 seropositivity in relation to age, being higher in the youngest age group 18–34 years (67%) and lower in the older age group (55–74 years, 59%, p trend = 0.004, Table 1). By contrast, in males, there was an increased seropositivity of anti-HPV-16 IgG in relation to increasing age, from 42% in those aged 18–34 years to 61% in those aged 65–74 years (p-trend = 0.003). Age-adjusted anti-HPV-16 IgG seropositivity was associated with an increasing number of sexual partners in females only, using the selected groupings (p-trend was 0.01 in females and 0.1 in males). Anti-HPV-16 IgG seropositivity was unrelated to level of education, place of birth (urban/rural), alcohol consumption (ever/never), tobacco smoking, or parity, age at first child birth, or use of hormonal contraception (data not shown for the last two variables). HPV-16 seropositivity was significantly higher in males resident in rural (67%), vs. urban areas (57%), (p = 0.05), but this association was not observed in females.

**Figure 1:**
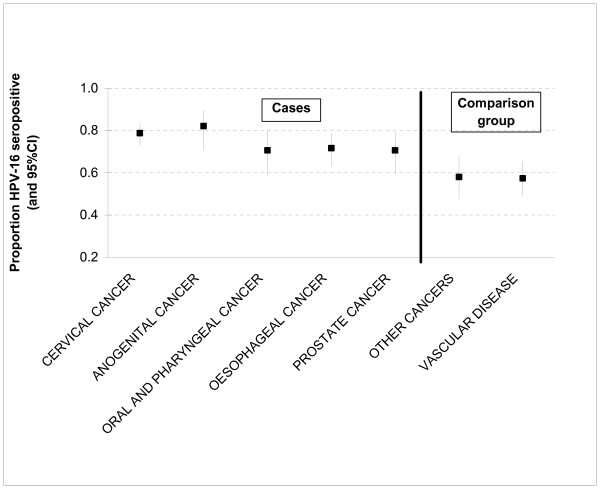
Adjusted proportions seropositive for HPV-16 IgG among the cases and comparison group.

Anti-HPV-16 IgG seropositivity in the comparison group was similar in those with vascular disease and those with the other cancers (Figure 1). Table 2 shows the odds ratios (OR) and 95% confidence intervals (95%CI) for the cancers of interest relative to the comparison group by anti-HPV-16 IgG antibody levels. An increasing trend in the risk of developing cancer of the cervix was found in relation to increasing anti-HPV-16 IgG levels; those with medium and high levels having adjusted odds ratios (OR) of 1.63 and 2.48 respectively (p trend <0.0001). Males and females seropositive against HPV-16 IgG antibodies were also at higher risk of developing cancer of other anogenital organs (OR = 2.50, p = 0.003 – the sample size was too small to divide the subjects with anogenital cancers into those with medium and high antibody levels). For cancer of the oral cavity and pharynx, subjects with medium and high anti-HPV-16 IgG levels had ORs (adjusted for age and sex) of 1.18 and 1.65, respectively (p trend = 0.05). However, after adjustment for potential confounding factors the odds ratios in relation to medium and high antibody levels were 1.09 and 1.47 respectively, p trend = 0.13). The risk for cancer of the oesophagus was found to be associated with increasing anti-HPV-16 IgG levels; adjusted odds ratios were 1.26 and 1.59 for medium and high antibody levels respectively (p-trend = 0.002). To test whether there was a differential effect of HPV -16 seropositivity and oesophageal cancer in males and females, we added an interaction term of sex by HPV -16 status into the logistic model; however this was not statistically significant (p = 0.63).

No statistically significant associations were found between anti-HPV-16 IgG antibodies and cancer of the prostate for either medium (adjusted OR = 1.34) or high anti-HPV-16 IgG levels (adjusted OR = 1.21, p-trend = 0.31).

## Discussion

### Case-comparison selection

This study is part of an ongoing cancer case control study run in the two major tertiary hospitals in Johannesburg, where the degree of diagnostic accuracy is high. Controls (comparison group) were selected from the pool of incident cancer patients and patients admitted for the fist time with vascular disease. In this study the controls were selected by excluding subjects whose cancer type was thought associated with the exposure of interest, e.g. those with squamous cell skin cancers and laryngeal cancers. We also excluded subjects with endometrial and ovarian cancer to avoid misclassification of the genital cancers of interest. Whilst a number of co-factors are thought to be important in the aetiology of the cancer types of interest (e.g. smoking), these were not part of the main hypothesis and so smoking related cancers were retained in the comparison group.

### HPV-16 serology

Virus-like particles (VLPs) engineered from various HPV types and used in enzyme-linked immunoassay (ELISA) have been validated as type specific antigens to detect serological markers of past and present HPV infection [23] Studentsov et al[18] indicated a twofold increase in sensitivity and specificity of the serological detection of anti-HPV-16 IgG antibodies using a polymer enhanced HPV-16 VLP ELISA protocol compared to previous HPV16 VLP ELISAs. As well as the improved detection of anti-HPV-16 IgG antibodies, the polymer-based ELISA also detects cross-reactive responses from closely related types to HPV-16 of alpha species A9 (containing HPV types HPV -31, -33, -35, -52 and -58) [24]. Williamson et al [25] found that in Cape Town, South Africa, the prevalence of HPV genotypes in cervical cancers was 46% for HPV-16, 2% for HPV-18, 6% for HPV-31, and 6% for HPV-33. Pegoraro et al [26] examined cervical tumour tissue from Zulu speaking black women in Durban, South Africa and found prevalences of 47% for HPV-16, 14% for HPV-18 and 25% for other HPV types. In another South African study of women with cervical or pre-invasive cancer [27], the prevalence of oncogenic genotypes in cervical biopsies was: HPV-16: 47%, HPV-18: 14%, HPV-33: 10%, HPV-35: 2%, HPV-52: 2% and HPV-58: 1%. However, the prevalence of types other than HPV-16 is expected to be low as responses to species A9 types other than HPV-16 would be to minor VLP epitopes [28] and so the antibody response in this study would be predominantly due to HPV-16.

Viral persistency and high viral loads are associated with an increase in the risk of cervical and anogenital cancers[29-31]. Anti-HPV IgG VLP ELISA optical density (which is proportional to the amount of antibody present) was found to be a reliable marker of past exposure to the HPV type tested and an indirect marker of high HPV viral loads measured using PCR[18].

In this study, a number of measures were introduced to minimise laboratory variation and bias. These included: i) using individual sera from a group of local volunteers to assess the reliability of the assay at low, moderate and high anti-HPV 16 IgG optical density levels; ii) adding a group of children's sera as a cross check for negative control values; iii) randomly allocating cases and the comparison groups on each plate to minimise case control bias; iv) 'blinding' the laboratory so that the assays were performed without knowledge of the disease or demographic status of the subjects; and v) testing cancers that are known to be associated with HPV (cervix, anogenital) and those with an uncertain association (oral cavity and pharynx, oesophageal, prostate) using the same assay, under the same laboratory conditions.

Whilst there is no gold standard to evaluate whether the anti-HPV-16 IgG optical density cut-off levels truly reflect infection by HPV 16, a cut-off of 4 SD above that found among healthy children in a previous study (>0.45) seemed appropriate for the purposes of this study [19]. This is similar to the approach used by Wang et al [32], who used 5SD as an appropriate cut off point as a proxy of persistent HPV infection. Using tertiles of anti-HPV IgG optical density level yielded similar results. Approximately 60% of the subjects in the comparison group (63% in females, 58% in males) had values above the cut off level.

#### Demographic distribution of anti-HPV16 IgG

In this study, the anti-HPV 16 IgG seropositivity patterns observed in the comparison group generally concur with other studies, namely that females have a higher seropositivity or prevalence of anti-HPV 16 IgG (and higher mean OD levels) than males, despite the latter reporting a higher number of sexual partners [33,34]. Stone et al [34] suggested that, in the USA, the sex differences may be due to differences in immune response, or higher HPV clearance rates among males. Very little work has been done in Africa on the demographic distribution of HPV in men. In this study anti-HPV 16 IgG seropositivity was not significantly associated with lower educational level; however there was a significant association with rural residence in men but not in women. It has been speculated that women using hormonal contraception might be more susceptible to HPV infection [35] but we did not observe this (data available on request) nor did another recent local study[36], nor was such an association found in the systematic review by Green and colleagues [35].

#### HPV-16 in relation to different cancer types

After adjustment for a number of potential confounding factors, in keeping with other studies, women with medium and high anti-HPV-16 IgG OD levels showed increasing risks of developing cancer of the cervix. Similarly, women and men with cancers of other anogenital organs showed at least a doubling of risk in relation to anti-HPV-16 IgG positivity. Consistent with a recent multi-centre study [3], we observed a weak association between anti-HPV 16 IgG antibody levels and cancer of the oral cavity and pharynx, but the test for trend ranged from borderline significance in the initial model, adjusted for age and sex (p = 0.05) to non-significant (p = 0.13) when all the important confounding variables were retained, suggesting that some confounding by lifestyle may be important. In the multicentre study [3] a stronger association was found between HPV-16 and oral cavity and pharyngeal cancers, but the numbers in this study would be insufficient to draw proper comparisons. Various oncogenic HPV types including HPV-16 infect squamous epithelia [2]. Squamous cell cancer is the predominant oesophageal cancer type found in South Africa[1]. A number of PCR based studies have isolated various HPV genotypes from oesophageal tissues [6,37-39], however the epidemiological evidence of an association with HPV-16 has been unclear. In this study we found a significant association between increased anti HPV-16 seropositivity and cancer of the oesophagus.

By contrast, no association in relation to anti-HPV-16 IgG antibodies was observed for prostate cancer. The literature regarding the association with HPV and the prostate (or a co-related infection) has been inconclusive. For example Dillner et al[10] found an increased risk of prostate cancer in relation to HPV-16 and HPV18 but no association for HPV-33 and HPV-11. In a subsequent study by Lagergren et al [7] no such association was found between HPV 16 and 18, but a possible association was found for HPV-33.

## Conclusion

If there is indeed an association between HPV-16 and oesophageal and possibly also some oral cavity and pharyngeal cancers, then emerging HPV vaccines may also reduce, at least in part, the incidence of these leading cancer types.
